# Brain-machine interface based on transfer-learning for detecting the appearance of obstacles during exoskeleton-assisted walking

**DOI:** 10.3389/fnins.2023.1154480

**Published:** 2023-03-14

**Authors:** Vicente Quiles, Laura Ferrero, Eduardo Iáñez, Mario Ortiz, Ángel Gil-Agudo, José M. Azorín

**Affiliations:** ^1^Brain-Machine Interface Systems Lab, Universidad Miguel Hernández de Elche, Elche, Spain; ^2^Instituto de Investigación en Ingeniería de Elche - I3E, Universidad Miguel Hernández de Elche, Elche, Spain; ^3^The European University of Brain and Technology (NeurotechEU), European Union; ^4^Biomechanics Unit of the National Paraplegic Hospital, Toledo, Spain; ^5^ValGRAI: Valencian Graduated School and Research Network of Artificial Intelligence, Valencia, Spain

**Keywords:** BMI, stopping intention, exoskeleton, EEG, transfer-learning, closed-loop

## Abstract

**Introduction:**

Brain-machine interfaces (BMIs) attempt to establish communication between the user and the device to be controlled. BMIs have great challenges to face in order to design a robust control in the real field of application. The artifacts, high volume of training data, and non-stationarity of the signal of EEG-based interfaces are challenges that classical processing techniques do not solve, showing certain shortcomings in the real-time domain. Recent advances in deep-learning techniques open a window of opportunity to solve some of these problems. In this work, an interface able to detect the evoked potential that occurs when a person intends to stop due to the appearance of an unexpected obstacle has been developed.

**Material and methods:**

First, the interface was tested on a treadmill with five subjects, in which the user stopped when an obstacle appeared (simulated by a laser). The analysis is based on two consecutive convolutional networks: the first one to discern the intention to stop against normal walking and the second one to correct false detections of the previous one.

**Results and discussion:**

The results were superior when using the methodology of the two consecutive networks vs. only the first one in a cross-validation pseudo-online analysis. The false positives per min (FP/min) decreased from 31.8 to 3.9 FP/min and the number of repetitions in which there were no false positives and true positives (TP) improved from 34.9% to 60.3% NOFP/TP. This methodology was tested in a closed-loop experiment with an exoskeleton, in which the brain-machine interface (BMI) detected an obstacle and sent the command to the exoskeleton to stop. This methodology was tested with three healthy subjects, and the online results were 3.8 FP/min and 49.3% NOFP/TP. To make this model feasible for non-able bodied patients with a reduced and manageable time frame, transfer-learning techniques were applied and validated in the previous tests, and were then applied to patients. The results for two incomplete Spinal Cord Injury (iSCI) patients were 37.9% NOFP/TP and 7.7 FP/min.

## 1. Introduction

Assistive walking devices are one of the technologies with the greatest projection for the rehabilitation of patients with severe motor dysfunction. The scientific community is designing frameworks for the more effective validation and control of these devices in the clinical setting. One alternative for improving the control of these devices is to actively involve the patient in the control. In this sense, neuroscience has much to say about how to close the motor intention-action loop (Ortiz et al., 2021). The field of brain-machine interfaces (BMI) is an emerging area, which is responsible for developing technologies for processing brain signals and establishing a connection between these signals and assistive devices. In practice, most BMIs use electroencefalographic (EEG) signals to record brain activity due to its relatively low cost, ease of use, and non-invasiveness (Wada et al., 2019). This technique uses electrodes placed on the scalp to record the electrical activity of superficial neurons (primarily the pyramids of the cortex), reflecting ongoing brain processes with exceptional temporal resolution (Bamdad et al., 2015). However, EEG also has important disadvantages as, for example, its low spatial resolution (Bamdad et al., 2015). Furthermore, it is highly sensitive to a wide range of artifacts, such as muscle movements, eye movements, or cardiac activity (Bamdad et al., 2015). Therefore, EEG is an extremely complex signal and with a high noise-to-signal ratio, making the direct decoding of individual brain processes particularly difficult to carry out. Nevertheless, a control signal can be used: visual potentials, within these steady-state visual evoked potentials (SSVEP), P300 potential, and error-related potentials (ErrPs). These potentials are quite identifiable in the average of the EEG as it mitigates the variability due to the noisy of the single-trial. However, designing robust classification paradigms that are capable of neglect this variability in the single-trial analysis remains a challenge within the field of BCIs (Blankertz et al., 2002). A large number of studies have addressed this problem by improving pattern recognition algorithms for ERP detection (Lotte et al., 2007). Methods based on linear discriminant analysis (LDA) (Salazar-Varas et al., 2015; Elvira et al., 2019) and support vector machines (SVM) are the most classic approaches (Rakotomamonjy and Guigue, 2008) achieve reasonable performance, especially when a large number of training examples are available. Bringing these paradigms to their ultimate goal, that is, real-time control, is a field in which literature works are starting to arrive. Applying these algorithms in the control of devices, such as exoskeletons, is a challenge, especially in terms of reliability.

In literature, the control of exoskeletons using exogenous potentials has been studied for command control, such as SSVEPs, which requires some voluntary control.

In Kwak et al. (2015), an asynchronous BMI based lower limb exoskeleton control system using SSVEPs was developed by decoding EEG signals in real time. The system used a visual stimulation unit with five LEDs fixed to the exoskeleton and a canonical correlation analysis method for frequency extraction.

One of these evoked potentials, the ErrPs, could be combined with interfaces like this. This way, if a potential is detected in response to an unexpected obstacle, the assistive device would stop. In this sense, there are works in the literature that address this issue, but not yet in real time (He et al., 2018). For example, classic machine learning algorithms trained with a considerable number of repetitions, common spatial patters (CSP) (Salazar-Varas et al., 2015) and a series of temporal features extracted from the signal (Elvira et al., 2019) were tested. In this type of application, a relevant factor to consider is the amount of false positives that could be generated in a real-time test. Of these works, the latter is the one that brings the paradigm closest to a realistic operation. However, aspects such as the number of repetitions and the increase of noise-to-signal ratio when having an assistive device such as an exoskeleton, must be taken into account to reformulate the techniques to be applied (López-Larraz et al., 2016). To reduce some of these problems, alternatives are arising that have been gaining more relevance in the last decade, such as neural networks (Zhang et al., 2020).

In Bellary and Conrad (2019), a deep convolutional network architecture using batch normalization and dropout layers was proposed to classify ErrPs elicited when the user was asked to monitorize the behavior of external events during EEG recordings with an accuracy of 79.2 ± 5.3%. In Usama et al. (2021), the objective was to classify single-trial ErrPs produced by individuals with stroke, investigate test-retest reliability, and compare different classifier calibration schemes with different classification methods such as Artificial Neural Network (ANN) and Linear Discriminant Analysis (LDA) with waveform features as input for meaningful physiological interpretability. The results showed that user and session specific calibration was needed for optimal decoding.

Specifically, transfer-learning techniques and fine-tuning help with the problem of not having a large amount of subject-specific data (Zhang et al., 2020). The great abstraction power of these algorithms is utilized to combine data from several subjects and extract descriptive features of the process. This technique has been discussed in some works in the literature. In Fahimi et al. (2019), a framework using a deep convolutional neural network (CNN) to detect attentive mental state from multi-channel raw EEG data was proposed. The approach included developing an end-to-end deep CNN to decode attentional information. Two strategies were tested during inter-subject transfer learning. The first one used a leave-one-subject-out strategy where a generalized network was learned using data from a pool of subjects and then the learned knowledge was transferred to a new subject reaching an accuracy of 79.1 ± 7.6%. The second one tried a subject adaptation approach that addressed the issue of information change/shift when transferring knowledge from the source to the target by retraining on a small sample size of the new subject's data, showing an increase of accuracy to 89.3 ± 4.4%.

Despite the fact that fine-tuning allows to reduce the number of repetitions needed by a subject for an acceptable accuracy, it still requires data from other subjects. This problem results in an endemic issue in the field: the difficulty of obtaining large amounts of high-quality data from subjects, with a minor content of artifacts and with a high user engagement, which in many cases results in a low performance of the application.

The objective of this study is to explore how to create an effective exogenous BMI model based on convolution networks that can be applied in the clinical field, with a reduced calibration time and sufficient efficacy for the subject to perform multiple repetitions. The BMI designed in this study aims to detect the user's EEG response to the intention to stop at an unexpected obstacle. The final purpose of the study is that the BMI detects the user's stopping activity and sends a command to an exoskeleton to close the control loop. For the development of the model, a novel approach is chosen that consists of two validation networks, to try to make the decoding more solid and reliable during sustained periods of time. The model is validated first on treadmill and then with control tests with a lower limb exoskeleton. This paper shows how a two-network approach is able to capture the changing dynamics of the EEG signal and how, by using transfer-learning models, generic data could be applied to specific user models reducing the number of repetitions while still obtaining acceptable results. The model was tested in non able bodied patients. This first validation step would help future studies to be able to scale these systems with more data and to increase consistency.

## 2. Materials and methods

In this work, the materials used for the conduct of tests, both treadmill tests and close-loop exoskeleton tests, are presented. The protocol followed by the subjects for the tests on the treadmill and exoskeleton is discussed. The methods used for preprocessing the EEG signal are showed, as well as the methodology used for training the two NN1 and NN2 classifiers that are responsible for encoding and decoding the EEG signal. Additionally, the metrics used for evaluating the models in both pseudo-online and online evaluations are explained. Finally, the way in which fine tuning can improve the model's performance and its application to the BMI model for tests with patients is discussed.

### 2.1. Materials

**Figure 1:**
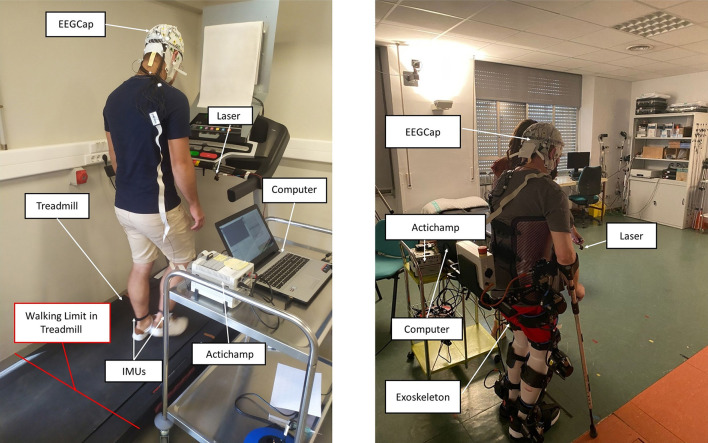
Treadmill setup for healthy subject **(Left)**; and setup with exoskeleton for healthy subject and non-able bodied patients **(Right)**.

The experimental setup is shown in Figure 1. For the electroencephalographic (EEG) recording, an actiCAP cap (Brain Products GmbH, Germany) with 27 electrodes was utilized. The electrodes followed the 10-10 distribution of the international system (Fz, FC5, FC1, FCz, FC2, FC6, C3, Cz, C4, CP5, CP1, CP2, CP6, P3, Pz, P4, PO7, PO3, PO4, PO8, FC3, FC4, C5, C1, C2, C6, CP3, CPZ, CP4, P1, P2, and POz). Four electrodes were placed for recording electrooculography (EOG). The reference was placed on the left earlobe (A1) and the ground on the right earlobe (A2). The EEG signal was recorded at 500Hz and amplified using the actiCHamp equipment (Brain Products GmbH, Germany). A hardware notch filter at 50Hz provided in the brain product equipment mitigated the network contribution. Hardware also filtered the signal over 0.1Hz to mitigate the DC component.

First data analysis used a Performance 750 treadmill (Pro-form) and a laser line projected in front of the treadmill, with a wavelength of 635 nm (red color) and an output power of 3 mW to warn the subject about the obstacle appearance. To detect the actual subject's stop, three inertial measurement units (IMUs) (WIT Motion, China) placed at head, left foot, and right foot were used. For each sensor, the accelerations on each of the axes: “Acc X”, “Acc Y”, and “Acc Z” were used for analysis. They collected the information at a 100Hz pace. The synchronization of the EEG, IMUs, and laser activations was managed by a custom software developed in Matlab (MathWorks Inc., Massachusetts, USA).

The H3 lower limb exoskeleton (Technaid, Madrid, Spain) was used for the second group of experiments, which also included real-time closed-loop control. The H3 is a walking exoskeleton with 6 degrees of freedom where hip, knee and ankle of each leg are motorized joints. The communication between the BMI and the H3 was done through a Bluetooth port. The connection was established on an Intel Core i7 laptop computer using MATLAB software.

### 2.2. Subjects

The study was conducted in accordance with the guidelines of the Declaration of Helsinki and was approved by the Institutional Review Board of Miguel Hernández University of Elche (DIS.JAP.03.18, 22/01/2019). Informed consent was obtained from all subjects involved in the study. The ethics committee consents to the exposure of images as long as the face is pixelated or indistinguishable.

Five healthy individuals were used to set up the model on a treadmill of which two were women and three were men, the average age is 24.8 ± 1.8. These individuals are referred as Treadmill Subjects ST.X, where X is the number of the subject.

Three healthy individuals were used for exoskeleton registration of which two were women and one was men, the average age is 25.7 ± 2.3. These individuals are referred as Exoskeleton Subjects SE.X, where X is the number of the subject.

The healthy subjects reported no diseases and they participated voluntarily in the study by giving their informed consent according to the Helsinkin declaration.

Two patients took part in the experiments at the National Hospital of Paraplegics of Toledo (Spain) over a period of 2 days, with ages of 21 and 55. These patients are referred as Exoskeleton Patients PE.X, where X is the number of the referred patient. The different session days are referred as PE.X Dn, where n is the first or second day.

### 2.3. Experimental procedure for registration

#### 2.3.1. Treadmill experiments

The subjects performed nine trials. Each trial had a duration of 120 seconds, during which the laser line was projected for one second with a random time between successive stimuli between 10 and 12 s. The total number of lasers per trial was nine which provides a total number of 81 repetitions.

The test began with the treadmill stopped for the IMUs calibration. Subjects remained steady and relaxed during the first 15 s for the convergence of the eye artifact elimination algorithm (Kilicarslan et al., 2016), then the treadmill was activated and the subject started walking at a constant pace of 2 km/h. When the subject was walking without holding on to the machine and at stable way, the 120 s trial was initiated.

**Figure 2:**
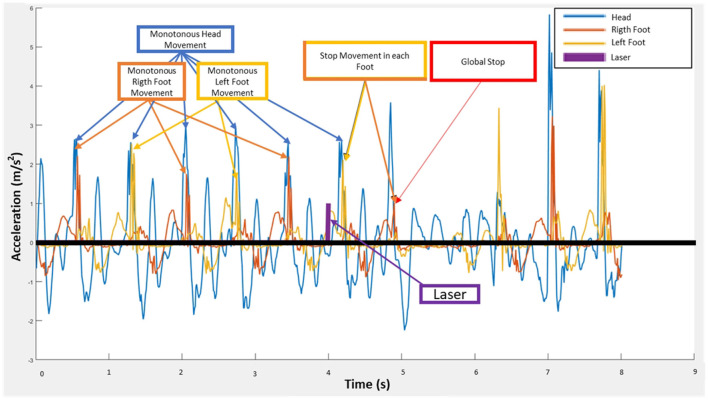
IMU stop detection based on the analysis of the right and left foot: Trial 2, Repetition 1, Subject SE.1.

The moment when the subject stopped was detected by analyzing the acceleration of two IMUs positioned on each foot. The signal from each IMU was preprocessed as follows: the modulus for the three axes of the accelerometer was taken, this signal was then filtered and the absolute value of the signal was taken. A peak detection algorithm (findpeaks in Matlab) was applied to this signal and peaks above a threshold of one were chosen. The first peak was chosen before two seconds after the laser appeared. The algorithm marked this moment for the left and right foot IMUs and chose the last point from these two in the timeline (see Figure 2). All repetitions were reviewed by a technician and any that were incorrectly labeled by the algorithm were corrected according to the specified criteria. The labeled moment was resampled from IMUs frequency to EEG frequency (100–500 Hz).

#### 2.3.2. Exoskeleton experiments

The EEG recording configuration was common with the treadmill setup. However, the treadmill was substituted by the H3 exoskeleton (Technaid, Spain). IMUs were not necessary as the exoskeleton provided the stop information and performed the function of tracking movement that the treadmill previously did (see Figure 1 right).

The repetition blocks of walking/obstacle were reduced due to the characteristics of commanding the exoskeleton, which is more demanding than a treadmill. Each healthy subject performed 8 trials. Each trial had a duration of 120 s, and the laser appearance was controlled in a similar way (1 second duration every 10–12 s). However, a period of four seconds after each activation of the exoskeleton was discarded to assure a stable exoskeleton walking which reduces the number of repetitions per trial to 5. The total amount of repetitions were 40.

As patients are more prone to fatigue, the number of trials was reduced to 4 and the randomized interval between laser repetitions was reduced to 6–8 s, obtaining 20 valid repetitions for patient.

During the close-loop tests, the subject remains at rest for 15 seconds. Then, the start signal is given and the exoskeleton starts walking. After that, at any moment the BMI can send an obstacle command and stop it. When this happens, the subject must help and not prevent the stopping. Exoskeleton then stops by the pre-established command and begins its preparation to start walking for the next laser repetition. During this second walking period which is the period of analysis, the BMI works without pre-established commands. This means that the subject must keep the output of the walking class during the walking period to avoid a FP. After the FP phase is passed, then the laser appears. At this moment the subject must try to stop the exoskeleton, if the model decodes the stopping intention correctly, the BMI outputs a stop command to stop the exoskeleton. ending the repetition. Then, the subject prepares for the next repetition. If the exoskeleton does not stop, a False negative is computed and the subject should continue, without impeding the walk, till the next laser for stopping appears corresponding to a new repetition.

### 2.4. EEG analysis

#### 2.4.1. Preprocessing

After the 0.1 Hz high-pass and 50 Hz filters applied by the brain products equipment, an ocular artifact filtering algorithm was applied to each of the 27 EEG channels (H∞, Kilicarslan et al., 2016), using the four EOG channels as reference (applied sample-by-sample). After that, four second-order filters by state variables, were applied to the H∞ filtered signal to obtain the following bands: 0.4–3 Hz, 2–4 Hz, 3–6 Hz, 5–8 Hz. Signals are given to the neural networks in these frequency bands.

**Figure 3:**
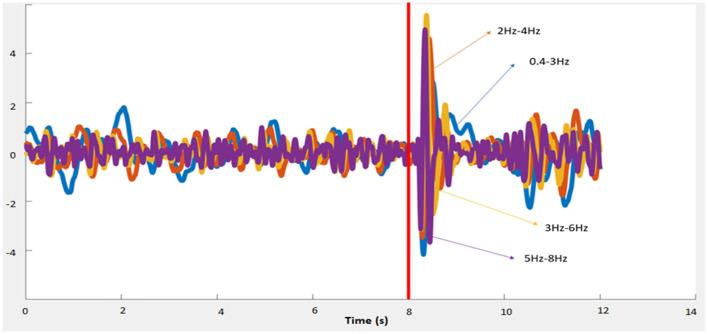
Average for all channels with different frequencies, in relation to the red laser event for subject SE.1.

The moment marked by the laser appearance defines the data classes. Activity that occurs before the event marked as obstacle appearance is labeled as class 1, referring to the walking moments prior to the obstacle appearance. Activity after the laser appearance and before the stop is labeled as class 2 which indicates the activity related to the obstacle stimulus and stopping. In this period, P300 intention potentials, error, and visual potentials may overlap in different frequencies and locations (see Figure 3). The classifiers employ epochs with a length of 0.6 s shifted at a 0.1 s pace.

#### 2.4.2. Network classifiers

The BMI developed looks for achieving a low FP/min ratio without comprimising the correct detection of the stopping. This critical aspect has led this work to approach a classification training model that is slightly different from the current state-of-the-art by introducing a novel way of training BMIs for EEG tasks.

Two classifiers have been proposed, which use the network scheme outlined in **Figure 5**. The two classifiers have been designed with the same network and training parameters. The network structure has been inherited from the work presented in Altan et al. (2019) (see Table 1). The present study employs a network that comprises three convolutional layers with max-pooling. The first layer is dedicated to preserve the spatial characteristics of EEG signals. Subsequently, two traditional convolutional layers, two fully-connected layers, and a dropout layer are utilized. Batch normalization and rectified unit (ReLU) activation are applied following each convolution operation in the convolutional layers. Training parameters are summarized in Table 2. The networks were trained using a computer equipped with an 11th Gen Intel(R) Core(TM) i7–11800 H system, 16GB of RAM, and a 64-bit architecture.

**Table 1:** Table with the parameters of the NN1 and NN2 network.

**Layers**	***Kernel***	**Filter numbers**
Input	-	-
conv	64x2	6
Batch normalization	-	-
ReLu	-	-
Max-pool	1x2	6
conv	1x11	12
Batch normalization	-	-
ReLU	-	-
Max-pool	1x2	12
conv	1x10	12
Batch normalization	-	-
Relu	-	-
Max-pool	1x2	12
Dropouc	-	-
Fully connected	60	60
ReLU	-	-
Fully connected	2	2
softmax	-	-

**Table 2:** Table with the training parameters of the NN1 and NN2 network.

**Parameters**	**Value**
Optimización	*Stochastic gradient descent with momentum*
Impulse	0.9
Learning rate	0.01
L2 regularization	0.0001
Fall factor	0.1
Dropt period	10
Epoch number	500
Batch size	100

##### 2.4.2.1. NN1 model

**Figure 4:**
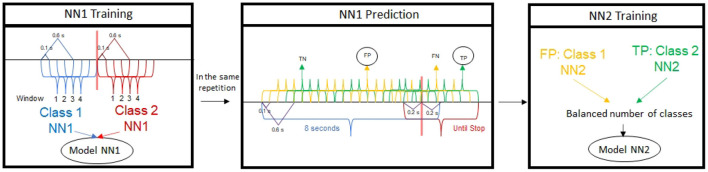
The training diagram for creating the two training models is as follows: in each training data package, the NN1 is first applied to a temporal window close to the Laser Obstacle, and then each epoch is predicted with the same repetitions over a wider temporal window. False Stops and True Stops are selected and balanced to be used in the NN2 model creation.

The first classifier (NN1) is trained with four epochs before and after the laser appearance for each repetition, i.e., 0.9 s before and after the stimulus were considered for Model NN1, see NN1 Training case in Figure 4. For the validation, the model is trained with N-1 trials using a leave-one-out cross-validation for the testing of the remaining trial, 8/1 for treadmill experiments and 7/1 or 5/1 for healthy and patient exoskeleton experiments.

##### 2.4.2.2. NN1 + NN2 model

The second classifier (NN2) acts as a corrector of the first classifier output. It is used during the pseudo-online and online analysis of the treadmill and exoskeleton experiments. To create this model, several steps are carried out using the training trials. For each of the N-1 trials used for the creation of the NN1 model, data is segmented as Class 1 for the epochs corresponding to the 8 s before the laser appearance, and as Class 2 for the seconds between the laser output and the actual stop, which is an undetermined time, but usually lower than 8 s. Notice, that due to the continuous time of analysis in pseudo and online analysis there are transition epochs. To determine the label of an epoch at least 0.4 of the 0.6 seconds must be in a class. As an epoch has a 0.6 s length, class 1 contains data from [-8,0.2] seconds and class 2 contains data from [-0.2, stop cue] seconds, being 0 the moment the laser is detected (see NN1 Prediction in Figure 4).

Each of the epochs of the training trials are then tested in the NN1 model, providing true (green epochs) or false (yellow epochs) detections for each class (see Figure 4 NN1 Prediction). The false detections (yellow epochs) of Class 1 correspond to False Positives (FP) and the true detections (green epochs) of Class 2 correspond to True Positives (TP). The NN2 model is then created using the data of the FP and the TP balancing the number of epochs for each class so both classes of the NN2 model have the same number of epochs (see NN2 Training in Figure 4).

**Figure 5:**
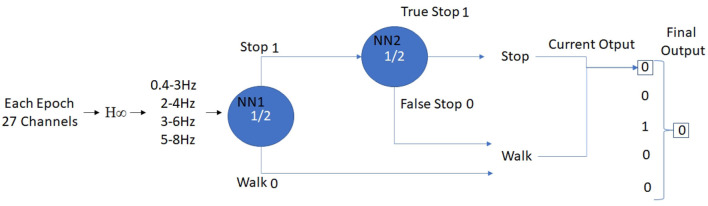
The prediction for the combination of NN1 and NN2 using pseudo-online (without output command) and online (with output command) is done as follows: each epoch is preprocessed with the described filtering stages, and each channel and filter is concatenated for input to the first network. This network can classify the epoch as walk or stop, that is 0 or 1. If it's a 0, it stops at the current output, but if it is a 1, the second classifier will check if it considers it a False Stop or a True Stop and in any case this value will pass to the current output. These values are accumulated and the Final Output is the mode of the last five outputs.

Once both models are created, testing trials are first classified by the NN1 model. If a Class 1 (Walking) is obtained as output, a walking command is registered by the BMI. However, if a Class 2 (Stop) is obtained, the epoch is then tested in the NN2 model to improve the BMI robustness. The NN1 model is corrected by the NN2 model, depending on the second classification as a false stop command (Class 1 of the NN2 model) or a true stop command (Class 2 of the NN2 model). In order to issue a stop command, at least 3 stop commands in 5 consecutive epochs must be registered. Figure 5 shows how the control of the output device is commanded by the BMI. In pseudo-online simulations the output is registered for validation purposes, while in online experiments is sent to the exoskeleton, providing the subject the close-loop feedback.

##### 2.4.2.3. Metrics for the assessment of the BMI performance

The validation of each of the models is carried out in a different way during pseudo-online and online analysis. During the pseudo-online validation the remaining training trial is tested in the system and the TP and FP/min are averaged following a leave-one-out cross-validation. For the online close-loop tests done with the exoskeleton, no cross-validation was done and the metrics correspond to the average results of the specific test trials, using all the training trials in the creation of the models.

The metrics assessed are: percentage of repetitions in which there were 0 FP and in the laser part there was at least one TP (NOFP/TP), percentage of repetitions in which there were no FP (NOFP) regardless of whether there was later a TP, percentage of repetitions that there was (TP) regardless of whether there was or not a FP, and finally, the number of FP per minute (FP/min).The FP/min metric gives an idea of how many FP there would be per minute, however it does not give an idea of how these are spread over the repetitions. Then, to assess how homogeneously the FPs are distributed, the NOFP metric gives an approximation of how many repetitions there are no FPs. Therefore, if this value is high but the FP/min are also high, it means that the FP were very accumulated in some repetitions. To evaluate how the FP affect the TP, i.e. how they would affect a real case, the NOFP/TP metric gives an idea of how many repetitions would have been totally correct if the test had been in real time.

For close-loop tests, the SuFP metric is added, which is the time it took for the BMI to fail, in those repetitions in which the BMI obtained a FP. In these tests the TP metric is only computed for those repetitions that passed the walking phase period.

### 2.5. Model validation with fewer repetitions: Fine-tuning

When using a BMI with non-able bodied subjects it is important to limit the time of experimentation as much as possible. However, this can affect the quality of the model. An optimization of the number of repetitions needed to create it becomes critical, as it has been reported that traditional machine learning algorithms are strongly affected by the number of repetitions used for the model training (Lotte et al., 2007).

The current research studies how the reduction of the number of training repetitions done by the subject affects the BMI performance. This can be mitigated thanks to the use of other subjects information through transfer learning (TL) (Goodfellow et al., 2016). The objective is to reduce the number of subject repetitions to 20, i.e. the number of repetitions conducted by non-able bodied subjects. The updated model uses the training trials of all the subjects (including the subject under analysis) and then it is fine-tuned (FT) using the 20 training repetitions of the subject under analysis.

To validate the approach, the results of the TL+FT model will be compared with the original model. As the objective is to develop a BMI capable to work in close-loop control in real time, there is not leave-one-out cross validation for the pseudo-online analysis. The original model is done by all the repetitions of the N-1 first trials. However, the TL+FL is first trained with all the training information of the rest of the subjects and with the first 20 repetitions of the first N-1 trials, and then, it is fine tuned with the same first 20 repetitions of the first N-1 trials. The results for each case are just computed for the last N trial. Table 3 shows the number of repetitions used for each model for the pseudo-online analysis comparison.

**Table 3:** Number of repetitions used for the comparison between the original model (NN1 + NN2) and the updated TL + FT model in the pseudo-online analysis.

		**Number of repetitions used**			
		**Original model**		**TL + FT model**	
**Experiment**	**Number of subjects**	**Training**	**Test**	**Training**	**Test**
Treadmill (9 trials)	5	72	9	4 x 81 + 20	9
Exoskeleton (8 trials)	3	35	5	2 x 40 + 20	5

The non-able bodied subjects were tested online using the exoskeleton, providing real-time feedback to the subject with the TL+FT model using the information of the 3 able-bodied subjects + the 20 repetitions of the training trials of each non-able subject in the day session. The number of testing repetitions was variable, depending on the fatigue shown by the subjects. Table 4 shows the number of repetitions used for the training and testing of the online BMI. The results were compared with the ones obtained by two able bodied subjects with the original model which number of repetitions are also shown in Table 4.

**Table 4:** Number of repetitions used by the BMI during its testing for the exoskeleton close-loop experiments with two able bodied subjects and two SCI patients.

		**Number of repetitions used**	
		**TL + FT model**	
**Experiment**	**Subject**	**Training**	**Test**
Able bodied exoskeleton	SE.1	40	30
	SE.3	40	35
SCI exoskeleton	PE.1 D1	3 x 40 + 20	20
	PE.1 D2	3 x 40 + 20	23
	PE.2 D1	3 x 40 + 20	27

Number of repetitions used for testing the original model (NN1 + NN2) in online experiments with real-time close-loop control of the exoskeleton with able bodied subjects. Only two of the three able bodied subjects performed the experiments. TL data corresponds to the 3 able bodied subjects + the 20 training repetitions of the SCI subjects in the session fine-tuned with them. Test repetitions are variable as the subjects were not able to complete the same number of repetitions due to their condition.

## 3. Results

Results' section is structured as follows. First subsection compares the performance of the NN1 model vs. the NN1+NN2 improved model for the treadmill and exoskeleton experiments with able bodied subjects. Second subsection provides the results of the two able bodied subjects that also tested the exoskeleton in close-loop control. Finally, third subsection shows the results of the two SCI patients that tried the TL+FT improved BMI with the exoskeleton in close-loop control.

### 3.1. Pseudo-online analysis of treadmill experiments in able bodied subjects

Table 5 shows a comparison of the performance of the BMI during a pseudo-online analysis of the treadmill data. Results are provided for the leave-one-out cross-validation of each subject (8/1 trials ratio). They compare the BMI performance when the classifier is only based on the NN1 model with the one that corrects the NN1 output with the NN2 classifier. The results of the Pseudo-Online with a single classifier are lower than those of the two-classifier methodology. Examining column by column, in average FP/min were reduced from 31.8 to 3.9. However, true positives (TP) were lower with the updated BMI, decreasing from 89.3% to 75.8%, making the BMI a bit more conservative. Nevertheless, repetitions with no FP increased from just a 38.8% to a 81.3%, while the repetitions with no FP and TP increased from 34.9% to 60.3%. As there is an improvement of the performance of the BMI based on the NN1+NN2 model the exoskeleton experiments were assessed using this combined model.

**Table 5:** Comparison of the performance of the BMI for treadmill experiments using NN1 model vs. NN1 + NN2 model.

	**Pseudo-Online NN1**				**Pseudo-Online NN1 and NN2**			
	**NOFP/TP**	**NOFP**	**TP**	**Fp/min**	**NOFP/TP**	**NOFP**	**TP**	**Fp/min**
	**(%)**	**(%)**	**(%)**		**(%)**	**(%)**	**(%)**	
ST.1	56.8	60.5	96.3	21.9	72.8	91.4	77.8	2.6
ST.2	40.7	43.2	95.1	31.0	81.5	91.4	88.9	2.9
ST.3	15.1	20.7	73.6	42.2	51.9	79.6	70.4	4.1
ST.4	33.3	37.5	91.7	29.9	45.8	65.3	76.4	5.2
ST.5	28.4	32.1	90.1	34.2	49.4	79.0	65.4	4.9
$\bar{x}$	34.9	38.8	89.3	31.8	60.3	81.3	75.8	3.9
σ	15.4	14.7	9.2	7.4	15.9	10.8	8.8	1.2

The results show per subject the average of the leave-one-out cross validation of the training trials.

### 3.2. Pseudo-online analysis of exoskeleton experiments in able bodied subjects

Table 6 shows the results of the combined model for the leave-one-out cross-validation of each subject during the exoskeleton experiments (7/1 trials ratio). In comparison with the treadmill, results show an increase in the FP/min and a lower ratio for the repetitions with NOFP and NOFP/TP.

**Table 6:** Performance of the BMI for exoskeleton experiments using NN1+NN2 model.

	**Pseudo-online NN1 and NN2**			
	**NOFP/TP (%)**	**NOFP (%)**	**TP (%)**	**Fp/min**
SE.1	32.5	67.5	55.0	14.3
SE.2	57.5	65.0	90.0	15.0
SE.3	50.0	62.5	77.5	11.6
$\bar{x}$	46.7	65.0	74.2	13.6
σ	12.8	2.5	17.7	1.8

The results show per subject the average of the leave-one-out cross validation of the training trials.

### 3.3. Close-loop control of exoskeleton experiments in able bodied subjects

Two of the able bodied subjects were able to fulfill several test trials in order to validate the behavior of the developed BMI in close-loop control. Data were registered and processed in real time, stopping the exoskeleton based on the online decoding of their brain signals. The model used included all the training registers. The results show the average metrics for the test trials carried out for each subject (see Table 7).

**Table 7:** Performance of the BMI working in close-loop control of the exoskeleton.

**Subjects**	**NOFP/TP (%)**	**NoFP (%)**	**SuFP (s)**	**TP (%)**	**FP/min**
SE.1	50.0	53.3	5.2	93.8	3.9
SE.3	48.5	54.3	4.7	89.5	3.7
$\bar{x}$	49.3	53.8	4.9	91.7	3.8
σ	1.1	0.7	0.3	3.0	0.2

Two of the three able bodied subjects completed the test trials.

The behavior of the BMI was more robust in the close-loop control than in the open-loop control represented by the pseudo-online analysis. Although, the ratio of the repetitions that did not present FP (NOFP) was a little lower, descending from 67.5% to 53.3% for SE.1 and from 62.5% to 54.3% for SE.3, the ratio of repetitions which had a TP with no FP (NOFP/TP) increased from 32.5% to 53.3% for SE.1 and kept a similar level 50.0% vs. 48.5% for SE.3. The number of FP was also lower, changing from 14.3 and 11.6 FP/min to 3.9 and 3.7 FP/min for SE.1 and SE.3 respectively. Notice that close-loop experiments really make the exoskeleton to stop and only compute a FP per repetition. Therefore, the NOFP/TP is a more fair index to compare the behavior of the online vs. the pseudo-online analysis. TP showed a more responsive behavior of the BMI, increasing from 55.0% to 93.8% for SE.1 and from 77.5% to 89.5% for SE.3. This fact makes it possible for the user to walk between 8 and 12 s without the interface stopping in at least 50% of the lasers. Individual metrics for each of the repetitions can be consulted in Supplementary material.

### 3.4. Reduction of the number of training trials. Analysis pseudo-online of the treadmill and exoskeleton experiments with able bodied subjects

In order to evaluate how the reduction of the number of training trials used affects the NN1+NN2 model classification performance, the data of the treadmill and exoskeleton able bodied subjects was assessed in a pseudo-online analysis for the last test trial. The trial was tested with models created with a different number of training repetitions. As it tries to represent the situation of an actual experiment, the first repetitions were included to include any possible influence of subject's lack of focus as the experiment goes on, and there was not a leave-one-out cross validation. In the case of the treadmill experiments, the number of repetitions used was 72, 40, and 20, and in the case of the exoskeleton experiments 35 and 20.

These results (see Table 8) show that all BMI indices are worse as the number of repetitions decrease. The objective is to diminish the number of repetitions to 20 for the experiments with non-able bodied subjects. However, the NOFP/TP ratio descends from 44.4% to 28.9% and the TP from 64.4% to 40.0% in the treadmill experiments. In the case of exoskeleton experiments as the reduction is just from 35 repetitions to 20 the decrease is lower, from 53.3% to 40.0% in the NOFP/TP ration and even increasing from 60.0% to 66.7% in the TP ratio.

**Table 8:** Comparison of the BMI performance depending on the number of training repetitions used for the model creation for the treadmill and exoskeleton experiments with able bodied subjects.

	**Number of repetitions for the model**	**Pseudo-online NN1 and NN2**			
		**NOFP/TP (%)**	**NOFP (%)**	**TP (%)**	**Fp/min**
Treadmill	72	44.4	71.1	64.4	8.5
	40	35.6	55.6	62.2	11.7
	20	28.9	66.7	40.0	18.7
Exoskeleton	35	53.3	66.7	60.0	14.0
	20	40.0	60.0	66.7	18.0

Results are averaged for the five treadmill and three exoskeleton subjects.

To mitigate this worsening, a transfer learning + fine-tuning model (TL+FT) was tested, following the best subject-adaptation model adopted in Fahimi et al. (2019). Table 3 shows the number of repetitions used for creating the fine-tuned model. This includes all the training repetitions of the rest of the subjects (81 repetitions x 4 subjects, treadmill, and 40 repetitions x 2 subjects, exoskeleton) and the first 20 repetitions of the subject under analysis using TL. After that the model is retrained with the first 20 repetitions of the subject (FT). Testing is carried out in the same way than in Table 8 using all the repetitions of the last trial.

The results of the TL + FT model shown in Table 9 improve the indices of the original treadmill and exoskeleton pseudo-online analysis. For the treadmill tests, the results improve increasing the NOFP/TP (44.4% vs. 55.6%) and NOFP (71.1% vs. 77.8%), as do the FPs which drop from 8.5 to 6.0 FP/min. For the exoskeleton results, the only index that does not improve is the NOFP/TP ratio (53.3% vs. 46.7%). This can be due to a casuistically bad distribution of the FP. Although the FP/min is lower, reducing from 14.0 to 2.0 and the ratio of repetitions without FP (NOFP) is also much more higher (66.7% vs. 86.7%).

**Table 9:** Analysis transfer learning for treadmill and exoskeleton model in healthy subjects.

		**Pseudo-online NN1 and NN2**			
		**NOFP/TP (%)**	**NOFP (%)**	**TP (%)**	**Fp/min**
Treadmill	72.0	44.4	71.1	64.4	8.5
	TL + FT	55.6	77.8	75.0	6.0
Exoskeleton	35.0	53.3	66.7	60.0	14.0
	TL + FT	46.7	86.7	60.0	2.0

### 3.5. BMI validation with non-able bodied subjects in close-loop control of the H3 exoskeleton

Once proved that the updated TL+FT model helps to improve the performance of the BMI, even though the number of training repetitions is dramatically reduced, the new model was tested in real-time close-loop control of the H3 exoskeleton with non-able bodied subjects. As only two non-able bodied subjects were tested, the TL was applied to the able bodied subject data, fine-tuning with the 20 training repetitions carried out by each non-able bodied subject. See Table 4 to check the number of repetitions used for training the BMI model. As the number of testing repetitions depended on the fatigue of the SCI patients, the number of test trials was variable, performing 20, 23 and 27 trials in the three sessions recorded. Comparing Table 10 with Table 7 it is clear that the BMI works in a more responsive way for the SCI subjects, showing a more sensitive behavior with an average of 100% vs. 91.7% ratio of TP and a NOFP/TP ratio of 37.9% vs. 49.3% for the three sessions. The average time since the start of the exoskeleton till a FP was computed was of 3.5±0.2 vs. 4.9±0.3 seconds. A higher FP index, even as it is not desirable, it is not such critical for a BMI that looks for a correct stop of a sudden obstacle, as it is more secure to have a higher TP at the cost of a higher FP value. Individual metrics for each of the repetitions can be consulted in Supplementary material.

**Table 10:** Performance of the BMI working in close-loop control of the exoskeleton.

**Subjects**	**NoFP/TP %**	**NoFP %**	**SuFP %**	**TP %**	**FP/min**
PE.1 D1	35.0	35.0	3.3	100.0	8.4
PE.2 D2	40.7	40.7	3.6	100.0	6.9
PE.1 D2	43.5	43.5	3.4	100.0	6.9
$\bar{x}$	37.9	37.9	3.5	100.0	7.7
σ	4.0	4.0	0.2	0	1.1

In the experiments two of the non-able bodied subjects complete the test trials.

## 4. Discussion

### 4.1. Discussion of the results

The main objective of the paper is to develop a BMI for stopping a exoskeleton when an unexpected obstacle appears preventing any possible risk of collision. As the BMI must be able to work in real-time, providing a close-loop control of the exoskeleton, all the analysis were developed with this in mind. Experimental data was first analyzed asynchronously in a pseudo-online study and validated after that in close-loop control with able bodied and SCI patients.

Results in the treadmill experiments demonstrate that a two step classifier (NN1+NN2 model) limits in a great way the number of FP (stops before the laser appears), even as it could make the system a little less responsive (lower TP). The comparison between the treadmill vs. exoskeleton experiments in the leave-one-out cross validation keep an acceptable TP ratio around 75%, but with an in increase in the indices associated with the FP. This is an expected outcome as it has been demonstrated that the difficulty to use a BMI working in combination with an exoskeleton is higher than with a treadmill (Ferrero et al., 2021). The two factors that can cause the worse performance with an exoskeleton are the existence of a higher noise-to-signal ratio and the difficulty to to keep a high level of focus on the motor task using the crutches. As Table 3 shows the number of repetitions used for creating the model was also much lower in the case of the exoskeleton experiments (72 vs. 35) which is another of the reasons of the inferior performance of the exoskeleton experiments in the pseudo-online results regarding the treadmill experiments. Nevertheless, as the objective of the BMI is to be able to stop when an obstacle appears, the capability of the tool to detect correctly an obstacle (TP) is a more crucial point than to keep an interrupted gait without undesirable stops (FP).

Close-loop control of the exoskeleton with the two able bodied subjects did not only keep the TP ratio of the pseudo-online analysis, but increased it to 91.7% without decreasing too much the ratio of repetitions that did not show a FP (53.8% vs. 65.0%). There are not many studies that test a BMI commanding an exoskeleton in real-time close-loop control (Ortiz et al., 2021), so even as a lower FP ratio is desirable, the performance of the BMI in real time presents an interesting case of study.

Other important contribution of the research focuses on the reduction of the time needed to perform a experimental session through the reduction of the number of training trials needed to create the model. This is accomplished using the information of other subjects through TL and fine-tuning the model with just 20 repetitions. The pseudo-online comparison in Table 9 shows that a reduction of the repetitions does not affect the TP, even increasing for the treadmill experiments. The FP indices are also improved, so a more robust BMI is presented. It is hard to establish a comparison with other literature works as they usually perform an offline analysis of the data, i.e. without simulating a real-time processing and validation of the signals. However, in order to compare the performance, an specific leave-one-out cross-validation for the accuracy in the identification of epochs of the two classes of model NN1 using TL+FT was computed. It showed a 89.9% accuracy which is similar to the 89.3% shown in Fahimi et al. (2019). Nevertheless, epoch accuracy of a classifier tested in an offline analysis does not give an accurate behavior of the possible behavior of the BMI in real time close-loop control and this index is given just as a comparison to other TL EEG-based literature works.

Finally, a validation of the BMI is presented in the research for two SCI patients completing 3 experimental sessions. Results show for the SCI patients a 100% TP ratio with a higher value of FP indices in comparison to the experimental tests conducted by able bodied subjects. It is hard to establish a pair to pair comparison as able bodied subjects did not use the TL+FT model for the close-loop tests. Generally, non-able bodied subjects show lower results than able bodied as they are more prone to fatigue. In fact, PE.2 was not able to complete the experimental tests in the first session, so only data from the second session is provided. However, the BMI was sensitive enough to assess a TP each time the patient was able to keep moving the exoskeleton till the laser appearance. This means that the SCI subjects were able to stop the exoskeleton quickly each time the laser appeared.

It can be affirmed that in the assembly of this work for exoskeleton we have obtained results superior to those of previous works, with traditional algorithms. Also taking into account that our algorithm has been applied with few repetitions per subject. Of the results that can be compared with other studies due to some differences in the assembly and number of repetitions: our study obtained a 60%TP and 2FP/min compared to the study of Salazar-Varas et al. (2015) whose extraction of characteristics was with CSP and the model was trained with LDA obtaining results of 30.0%TP and 6.7FP/min and the study of Elvira et al. (2019) whose extraction of characteristics were temporal and the model was trained with LDA obtaining results of 63.9%TP and 2.6FP/min.

If the literature examples of BMIs working in real-time close-loop control of an experiment are limited, those that work with non-able bodied are much more unusual (Ortiz et al., 2021). This makes hard to compare our SCI results with other works that study the EEG response to an obstacle with SCI patients.

### 4.2. Limitations of the study

As the number of subjects is limited, it is not possible to perform an statistical study to assure that the conclusions extracted are not due to the variation in performance of the subjects, which is a reported problem of EEG studies in other works (Ortiz et al., 2020, 2021). It is important to remark, that a lot of the experiments in literature are based on datasets acquired from one subject, and analyses based on more than 10 subjects are rare (Wierzga et al., 2018). Moreover, results even as they are given as a case of study, could be used for the comparison of other future studies.

The weaker point of the proposed close-loop control BMI is the limited number of repetitions the subject was able to keep the exoskeleton walking to reach the laser appearance. This value was in average 53.8% for able bodied subjects and only a 37.9% for SCI patients. Even though the most important index for the correct detection of the obstacle is marked by the TP, achieving a 91.7% for able bodied subjects and a 100% for patients. This means that the BMI is able to correctly stop the exoskeleton after the laser appearance with a high accuracy. However, a more comfortable BMI would require a reduction of the FP to avoid undesirable stops of the exoskeleton.

Future research should be focus on the reduction of the FP without compromising the correct detection of the laser. To decrease FP, the first step should be to increase the mode (five in this study) and observe the extent to which true positives (TP) are degraded. This could be done with an adjustment algorithm.

Exploring the nature of FPs and why they occur in bursts could help. They could be related to noise that may be increased by the location of the reference (in this study the ear), e.g. the reference at FCz may decrease noise due to motion artifacts. Studying the most appropriate reference could be a potential source of improvement, especially in movement-based BMIs.

Finally, increasing the sample size is always a consideration that could help to improve the BMI performance. However, this is one of the main challenges in the field of deep learning in BMIs. Obtaining specific data and conducting this type of testing involves a significant effort in terms of instrumentation and user involvement. An alternative that is gaining popularity in the field is to train the network on various tasks (a network that is capable of, for example, segmenting images very well) and then retrain it on the specific task [in this case EEG specific task, like obstacle reaction, Sadiq et al. (2022)], which could be another future approach to test.

In conclusion, in this work, a CNN model has been explored to create a BMI that, through two step classifiers, is able to distinguish the walking pattern of the evoked potential generated in the EEG when the user decides to stop due to an unexpected obstacle. It has been shown that the performance of the proposed BMI depends on the number of repetitions with which it is trained. The transfer learning model with fine-tuning was able to address this issue. The network has been tested on SCI patients with success and with acceptable percentages, even though a reduction of FP should be addressed in future works.

## Data availability statement

The datasets presented in this article are not readily available because of Miguel University of Elche Ethical Committee Review Board. Requests to access the datasets should be directed to EI, eianez@umh.es.

## Ethics statement

The studies involving human participants were reviewed and approved by Institutional Review Board of Miguel Hernnndez University of Elche (DIS.JAP.03.18, 22/01/2019). The patients/participants provided their written informed consent to participate in this study.

## Author contributions

Conceptualization and data curation: VQ and EI. Methodology: VQ, LF, ÁG-A, and EI. Software: VQ, LF, and EI. Validation: EI and MO. Formal analysis and investigation: VQ and LF. Resources: MO, EI, and JA. Writing—original draft preparation: VQ and MO. Writing—review and editing: VQ, MO, and EI. Visualization: VQ. Supervision: EI, MO, ÁG-A, and JA. Project administration: ÁG-A and JA. Funding acquisition: JA. All authors have read and agreed to the published version of the manuscript.
