# Supplementary material for: Brain-machine interface based on transfer-learning for detecting the appearance of obstacles during exoskeleton-assisted walking
Source: Front Neurosci. 2023 Mar 14;17:1154480. doi: 10.3389/fnins.2023.1154480 (PMC10043233; doi:10.3389/fnins.2023.1154480)
Supplement: Supplementary file 1 [file Data_Sheet_1.PDF]

Supplementary Material

1 SUPPLEMENTARY TABLES AND FIGURES

Table S1. All close-loop repetitions performed by each able bodied subject. In the Time before FP column, the time before an FP (in black) is displayed, the time the BMI remained without FP until the laser appeared and then stopped before two seconds (in green), and the time the BMI remained without FP until the laser appeared and then did not stop before two seconds (in red) is shown. - mark if the repetition fail

SE.1			SE.3		
Time before FP	Trial	Repetition	Time before FP	Trial	Repetition
5,2	1	1	4,2	1	1
8,4		2	11,2		2
5,8		3	10,8		3
12		4	6,2		4
8,2		5	12		5
2,3	2	6	4,9	2	6
8,3		7	3,2		7
12		8	8,3		8
8,4		9	7,1		9
3,7		10	3,9		10
8,9	3	11	8,2	3	11
5,9		12	4,1		12
8,2		13	11,7		13
9,3		14	9,2		14
5,1		15	9,3		15
3,4	4	16	10,3	4	16
8,9		17	6,3		17
8,1		18	9,2		18
6,3		19	3,6		19
11,4		20	8,2		20
8,2	5	21	2,1	5	21
6,3		22	8,1		22
4,1		23	4,9		23
8,1		24	5,8		24
5,2		25	8,9		25
8,4	6	26	9,6	6	26
5,9		27	7,9		27
5,8		28	4,9		28
8,2		29	10,2		29
7,2		30	6,3		30
			2,9	7	31
			9,4		32
			10,2		33
			4,2		34
			9,1		35

Table S2. All close-loop repetitions performed by each non-able bodied. In the Time before FP column, the time before an FP (in black) is displayed, the time the BMI remained without FP until the laser appeared and then stopped before two seconds (in green), and the time the BMI remained without FP until the laser appeared and then did not stop before two seconds (in red) is shown. - mark if the repetition fail

PE.1 D1			PE.2 D2			PE.1 D2		
Time before FP	Trial	Repetition	Time before FP	Trial	Repetition	Time before FP	Trial	Repetition
6,2		1	7,2		1	6,8		1
2,6		2	4,4		2	7,9		2
7,1	1	3	7,9	1	3	4,8	1	3
2,2		4	6,9		4	3,9		4
5,1		5	5,1		5	3,8		5
4,2		6	3,3		6	1,7		6
8		7	6,9		7	6,2		7
2,2	2	8	-	2	-	3,9	2	8
7,3		9	-		-	2,7		9
3,2		10	-		-	8		10
5,1		11	2,9		8	4,2		11
6,1		12	4,1		9	6,5		12
7,7	3	13	7,2	3	10	2,6	3	13
2,9		14	8		11	8		14
4,2		15	3,9		12	3		15
1,1		16	7,1		13	2,3		16
2,4		17	4,2		14	6,3		17
5,1	4	18	7,8	4	15	7,2	4	18
7,2		19	6,2		16	-		-
3,2		20	1,9		17	-		-
			3,1		18	6,2		19
			4,2		19	3,6		20
			2,2	5	20	3,8	5	21
			5,2		21	7,2		22
			4,8		22	3,9		23
			3,2		23			
			6,2		24			
			4,1	6	25			
			7,3		26			
			2,2		27			
